# Limb restraint induces a rapid and profound decrease in core body temperature associated with the stress response in mice

**DOI:** 10.1093/pnasnexus/pgag301

**Published:** 2026-09-04

**Authors:** Masashi Watanave, Rei Izumi, Momoka Aizaki, Takumi Hatai, Naoi Sakaguchi, Sakura Hayashi, Miho Ashikawa, Megumi Eguchi, Hayato Umehara, Shun Yamaguchi

**Affiliations:** Department of Morphological Neuroscience, Gifu University Graduate School of Medicine, 1-1 Yanagido, Gifu 501-1194, Japan; Department of Morphological Neuroscience, Gifu University Graduate School of Medicine, 1-1 Yanagido, Gifu 501-1194, Japan; Department of Morphological Neuroscience, Gifu University Graduate School of Medicine, 1-1 Yanagido, Gifu 501-1194, Japan; Department of Morphological Neuroscience, Gifu University Graduate School of Medicine, 1-1 Yanagido, Gifu 501-1194, Japan; Department of Morphological Neuroscience, Gifu University Graduate School of Medicine, 1-1 Yanagido, Gifu 501-1194, Japan; Department of Morphological Neuroscience, Gifu University Graduate School of Medicine, 1-1 Yanagido, Gifu 501-1194, Japan; Department of Morphological Neuroscience, Gifu University Graduate School of Medicine, 1-1 Yanagido, Gifu 501-1194, Japan; Department of Morphological Neuroscience, Gifu University Graduate School of Medicine, 1-1 Yanagido, Gifu 501-1194, Japan; Department of Morphological Neuroscience, Gifu University Graduate School of Medicine, 1-1 Yanagido, Gifu 501-1194, Japan; Department of Morphological Neuroscience, Gifu University Graduate School of Medicine, 1-1 Yanagido, Gifu 501-1194, Japan; Center for One Medicine Innovative Translational Research, Gifu University Institute for Advanced Study, 1-1 Yanagido, Gifu 501-1194, Japan

**Keywords:** restraint stress, hypothermia, limb restraint, brown adipose tissue, vasodilation

## Abstract

Stress responses are triggered by diverse stressors and typically involve acute common physiological changes including increased heart rate and adrenocortical hormone secretion. In contrast, core body temperature (Tc) may either increase or decrease depending on the stressor. Although restraint is widely used to induce stress responses in laboratory animals, heterogeneous effects on Tc have been reported. To elucidate the central and peripheral mechanisms underlying restraint-induced hypothermia, a reliable model that consistently induces hypothermia is first required. In this study, we aimed to establish an experimental system using mice that can reliably induce hypothermia through physical restraint. Furthermore, we aimed to elucidate the primary factors contributing to hypothermia within the various elements constituting the restrained state and to simplify the restraint experimental system by eliminating unnecessary elements. Starting with conventional cylinder-shaped restraint equipment and modifying it in various ways, we finally found that the simple procedure of limb restraint reliably induces a rapid (within 15 min) and substantial (∼4 °C) decrease in rectal temperature, a good indicator of Tc. Limb restraint increased serum corticosterone concentration and activated the paraventricular hypothalamic nucleus, suggesting that a type of stress response involving Tc reduction was induced by limb restraint. The decrease in body temperature caused by limb restraint was attributed, at least in part, to reduced heat production in the brown adipose tissue and increased heat loss from the tail. This simplified restraint paradigm provides a useful tool for elucidating the mechanisms underlying hypothermia induced by physical restraint.

Significant statement A few types of stressors are known to cause temporary hypothermia. This hypothermia, like the hyperthermia induced by many stressors, is considered a crucial survival strategy under stressful conditions, yet its underlying thermoregulatory mechanisms remain poorly understood. Restraint is a widely used paradigm for inducing stress responses in experimental animals, but it has been reported to sometimes cause hypothermia and sometimes hyperthermia, making it an inadequate model for investigating thermoregulatory mechanisms. In this study, using mice, we examined various components of restraint and ultimately determined that limb restraint is the most critical factor in inducing hypothermia. The limb restraint paradigm reliably induces rapid and significant hypothermia, making it an excellent platform for elucidating the mechanisms underlying restraint-induced hypothermia.

## Introduction

Stress responses are triggered by diverse stressors and typically involve acute common physiological changes, including increased heart rate, elevated blood pressure, increased arousal, increased muscle tension, and increased adrenocortical hormone secretion (1–4). However, Tc levels may either increase or decrease depending on the type of stressor. In laboratory animals, numerous stressors—such as psychological stressors like social defeat experiences (5, 6), exposure to novel environments (7, 8), and social isolation (9); or physical stressors like tail pinch (10) and foot shock (11)—have been reported to cause transient increases in Tc. This Tc elevation, along with increased blood circulation, is thought to enhance muscle and central nervous system performance in classic “fight-or-flight” situations, providing an advantage in overcoming critical circumstances (12, 13). On the other hand, relatively few types of stressors, such as hypergravity conditions (14), hypoxia (15), innate fear odor (in mice) (16), and some forms of restraint have been reported to cause a decrease in Tc (17–21). This decrease in Tc can be interpreted as a mechanism that conserves or efficiently utilizes energy in life-threatening situations in which the “fight-or-flight” response is ineffective, thereby conferring a survival advantage (13, 22–24). Therefore, the rise and fall of Tc associated with the stress response can be considered as two important alternative strategies for survival in stressful situations.

The mechanisms involved in these thermoregulatory processes, namely, the control systems for heat production and heat dissipation in the periphery and the neural circuits in the central nervous system that regulate them have been investigated in a limited number of cases, such as Tc elevation due to social defeat (12, 25), Tc reduction due to innate fear odors (26), and Tc reduction due to hypergravity (14).

However, despite restraint being one of the most commonly used paradigms for inducing stress responses in laboratory animals, the peripheral mechanisms and central neural circuits responsible for the resulting changes in Tc remain unclear. One major reason for this is that the change in Tc caused by the restraint is not constant. In fact, previous studies using restraint paradigms have reported not only decreases but also increases in Tc levels in restrained animals (13, 27–30). This is thought to be influenced by numerous factors, including the species, strain, and age of the experimental animals used, as well as the characteristics of the restraint equipment employed in the study.

In this study, we aimed to establish an experimental system using mice that can reliably induce hypothermia through restraint. Furthermore, we aimed to elucidate the primary factors contributing to hypothermia within the various elements constituting the restrained state such as restrictions on movement in various parts of the body, physical contact with the restraining apparatus, and confinement in closed spaces—and to simplify the restraint experimental system by eliminating unnecessary elements. Here, we demonstrate that limb restraint is a primary factor contributing to hypothermia and that limb restraint alone can reliably induce a rapid and significant drop in body temperature. Furthermore, we show that the hypothalamic-pituitary-adrenal (HPA) axis is activated during limb restraint, and that the decrease in body temperature is at least partially attributable to suppressed heat production in the brown adipose tissue (BAT) and increased heat loss from the tail.

## Materials and methods

### Animals

Male C57BL/6N mice (5- to 6-week-old) were purchased from Japan SLC Corporation (Hamamatsu, Japan) and used for experiments at 7-week-old. The mice were housed at 23 °C in an environment with a 12–12 h light–dark cycle (8 AM–8 PM: light phase) with food and water ad libitum for at least 1 week before the start of the experiments. The experimental protocol was approved by the Institutional Committee for Animal Research of Gifu University (Permission Number: 2021-278, AG-P-N-20250072). All procedures related to the care and treatment of animals were performed in accordance with the Guidelines for Animal Experiments of Gifu University and the Japanese Act on the Welfare and Management of Animals.

### Restraint procedure

Seven-week-old mice (20–25 g weight) were subjected to the experiments at 2–4 PM. Restraint experiments in which the mice were in a thermoneutral state were conducted at 30 °C, while all other restraint experiments were conducted at 23 °C. The mice were habituated to the experimental room and arena (white or brown boxes: 10 × 20 × 20 cm, or breeding cages with animal bedding: 20 × 30 × 15 cm) for 30 min per day for 3 days prior to the restraint experiment. On the day of the restraint experiment, the mice were habituated to the arena for 30 min, after which they were exposed to various restraint conditions for 15–75 min. As a control for the acrylic equipment restraint, the mice were sham-operated by momentarily placing them in similar acrylic cylinders or boxes and then releasing them. In some experiments, the mice were restrained using glue traps (Nezumi Hoihoi Chu-bai-chu, Earth Corporation, Tokyo, Japan). In another experiment, the limbs or abdomen were fixed to cardboard (100 × 100 mm, Material Research Center, Kawasaki, Japan) using cyanoacrylate adhesive (Toagosei Company, Tokyo, Japan). As a control for this experiment, a 5 × 5 mm cardboard piece was attached to each limb using the same adhesive.

### Acryl apparatus for restraint

All acryl apparatuses of various shapes used in the present study were generated by Sakura Plastics Company (Yokohama, Japan). The mice were restrained using transparent acryl cylinders (63 mm length) of various inner diameters (25, 30, 35, 40, and 45 mm). All cylinders had a 5 mm wide slit in their upper part to enable the measurement of the body surface temperature using a thermographic camera. The cylinders also contained air holes (Φ = 2 mm) at 5 mm intervals in their front walls and at 10 mm intervals in their cylindrical walls to allow the mice to breathe normally. The 25 mm diameter cylinder was remodeled in some experiments to have 6 mm width slits on both sides to allow the mice to extend their limbs out of the cylinder. In some experiments, the mice were restrained in rectangular acrylic boxes. The inner dimensions (length **×** width **×** height) of the boxes were 63 **×** 53 **×** 30, 63 **×** 45 **×** 35, 63 **×** 30 **×** 53, and 63 **×** 35 **×** 45 (mm), each with a volume of ∼100 mL. They also contained slits of 5 mm width in their upper part and air holes (Φ = 2 mm) in their multiple vertical walls.

### Measurement and analysis of body surface and rectal temperatures

Thermal images of each animal were recorded every 5 min using an infrared thermal imaging camera (FLIR3; Teledyne FLIR LLC, Wilsonville, OR, USA). The camera was set at a height of 20 cm and the radiation ratio was 0.95. The body surface temperature was obtained from thermal images using a software package (FLIR Tools). The highest temperature in the area near the midline of the back was represented as the body surface temperature. During cylindrical restraint, the surface temperature was measured by ensuring that the area near the midline of the back was visible through the cylinder's slit. To investigate peripheral heat production and heat dissipation, we measured the surface temperature of the skin covering the interscapular BAT (iBAT) and the tail. To measure iBAT temperature, the back hair was shaved under anesthesia with isoflurane 1 day before experiments. The tail temperature was measured at the base of the tail. The rectal temperature of the mice was measured using rectal thermometer probes (Natsume Seisakusho, Tokyo, Japan) at 15, 60, or 75 min after the start of restraint. The probes were gently inserted 1 cm into the anus, and the rectal temperature was measured.

### Measurement of serum corticosterone concentration

The mice were subjected to limb restraint for 30 min. The mice were then decapitated to collect trunk blood. The blood samples collected in tubes were immediately centrifuged at 4 °C at 1,700 rpm for 15 min, and the supernatant was collected as the serum. Serum corticosterone levels were measured using an ELISA kit (Yanaihara, Shizuoka, Japan) according to the manufacturer's instructions.

### Immunohistochemistry

Immunohistochemistry was performed as previously described (31) with minor modifications. Briefly, 2.5 h after limb restraint, the mice were deeply anesthetized and transcardially perfused with phosphate-buffered saline and 4% paraformaldehyde (in 0.1 M phosphate buffer), and 50-μm-thick coronal brain slices were obtained. The coordinates of the slices were identified in accordance with Paxinos and Franklin's the Mouse Brain in Stereotaxic Coordinates (4th edition). The tissue slices were treated with the following primary antibodies: guinea pig monoclonal anti-c-Fos (1:1,000; Gp108B5 (226308); Synaptic Systems, Goettingen, Germany) or mouse monoclonal anti-NeuN (1:500, MAB377; Merck, Darmstadt, Germany) and the following secondary antibodies: Alexa Fluor 488-conjugated donkey antiguinea pig IgG (1:500; Jackson Immuno Research, West Grove, PA, USA), or Alexa Fluor 647-conjugated donkey antimouse IgG (1:1,000; Thermo Fisher Scientific, Waltham, MA, USA). NeuroTrace 640/660 (1:500; Thermo Fisher Scientific) was used for Nissl staining. Fluorescence images were obtained using a fluorescence microscope (BZ-X810; Keyence, Osaka, Japan), and the density of c-Fos positive cells was quantified using the structured illumination microscopy mode. Subsequently, the cells were counted using a BZ-Analyzer (Keyence, Osaka, Japan) and divided by the area of the region of interest (ROI).

### Drug administration

RU486 (Selleck Chemicals, Houston, TX, USA) dissolved in dimethyl sulfoxide (DMSO) at a concentration of 100 μg/μL was diluted 10-fold with sesame oil (Sigma-Aldrich, St. Louis, MO, USA). Mice were intraperitoneally injected with 200 μL of RU486 solution (2 mg/mouse) (32), the vehicle (DMSO and sesame oil, 1:9 v/v), or sesame oil alone. One hour after the injection, the mice were subjected to limb restraint.

### Statistical analysis

Statistical analyses were performed using the Welch test, Bonferroni post hoc test after one-way ANOVA, repeated measures ANOVA, or two-way ANOVA, and Dunnett's test following Welch's ANOVA with GraphPad Prism 9 software (GraphPad Software, San Diego, CA, USA). Data are expressed as mean ± SEM.

## Results

### Body temperature reduction was induced by restraining the mice in acrylic cylinders of a specific diameter range

**Figure 1:**
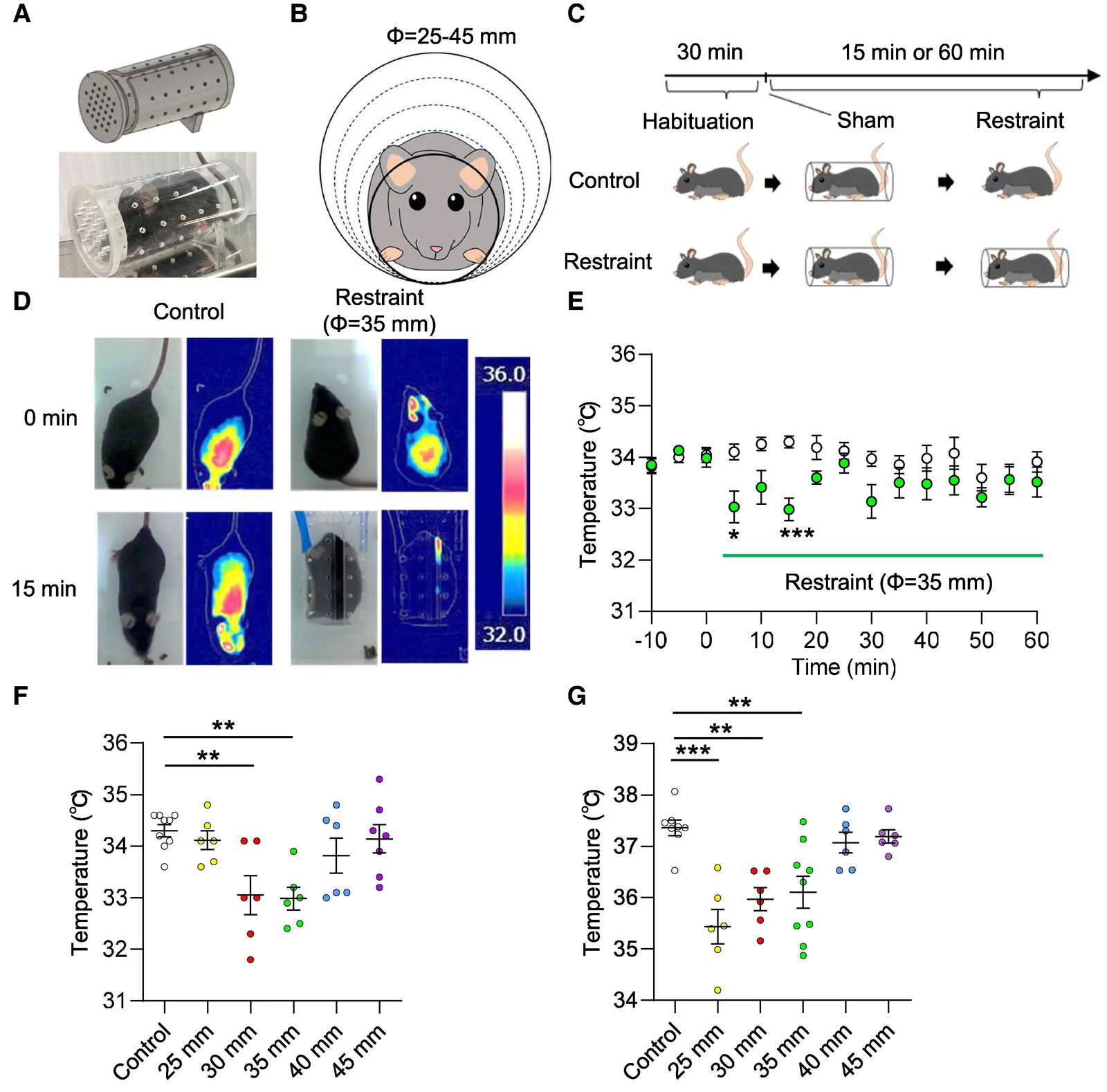
A decrease in body surface temperature and rectal temperature was induced by restraint using an acrylic cylinder of a specific diameter range. A) Acrylic cylinders of various diameters were used to restrain the mice. The length of the cylinders was 63 mm, and they contained many Φ = 2 mm holes in the front and cylindrical walls. The photograph of a mouse restrained with Φ = 30 mm cylinder is shown on the lower side. B) Schematic illustration of a mouse and cylinders with diameters ranging from 25 to 45 mm in 5 mm increments. C) Schematic of the experimental schedule. The mice were restrained for 15 or 60 min after 30 min of the habituation period. D–F) Body surface temperature was monitored using a thermograph camera. Representative images of control mice and the mice restrained with a 35 mm diameter cylinder just before (0 min) and 15 min after restraint or sham operation are shown in (D). The body surface temperature was measured every 5 min. The mice were restrained or sham-operated at time = 0. The time plot of the control condition and mice restrained with Φ = 35 mm cylinder is shown in (E). The body surface temperature of each restraint condition at 15 min are shown in (F). G) Rectal temperatures were measured at 15 min. Statistical differences were determined using the following tests: Bonferroni's test following Repeated measures ANOVA in (E), and Bonferroni's test following one-way ANOVA in (F, G) (*P < 0.05, **P < 0.01, ***P < 0.001).

In previous studies employing the restraint stress paradigm, mice of various ages and strains and a variety of restraint devices have been used (21, 33–36). We used 7-week-old C57BL/6N male mice weighing 20–25 g and acrylic cylinders for restraint. To examine the effect of cylinder diameter, we used cylinders with diameters ranging from 25 to 45 mm in 5 mm increments (Fig. 1A and B). When a mouse was placed in a 30 mm diameter cylinder, there was almost no gap between the lateral part of the mouse's body and the inner wall of the cylinder. When placed in a 25 mm diameter cylinder, the mouse's body was slightly compressed by the cylinder wall. The inner length of the cylinders was 63 mm, which was sufficient to fit a mouse from the tip of its nose to the base of its tail. The cylinders had a 5 mm wide slit at the top for measuring the body surface temperature with a thermographic camera, and many holes (Φ = 2 mm) in their front and cylindrical walls to maintain breathable conditions for the mice.

After habituation to the experimental arena for 30 min, the mice were restrained in cylinders of various diameters for 15 or 60 min (Fig. 1C). The body surface temperature was monitored using a thermographic camera (Figs. 1D, E and S1). As shown in Fig. 1E, mice placed in a 35 mm diameter cylinder temporarily exhibited a significant decrease in body surface temperature compared with controls (Control _(5min)_: 34.10 ± 0.15 °C, *n* = 9, 35 mm _(5min)_: 33.03 ± 0.31 °C, *n* = 6, *P* = 0.014; Control _(15min)_: 34.30 ± 0.12 °C, *n* = 9, 35 mm _(15min)_: 32.98 ± 0.22 °C, *n* = 6, *P* < 0.001), which then recovered largely by 60 min. Compared with the control group at the 15-min time point, a significant decrease in body surface temperature was also observed with the 30 mm diameter cylinder (30 mm: 33.05 ± 0.38 °C, *n* = 6, *P* = 0.005) but no significant difference was seen with the 25-, 40-, or 45-mm cylinders (25 mm: 34.12 ± 0.18 °C, *n* = 6, *P* > 0.999; 40 mm: 33.82 ± 0.34 °C, *n* = 6, *P* = 0.841; 45 mm: 34.14 ± 0.28 °C, *n* = 7, *P* > 0.999).

We further recorded the rectal temperature at 15 min to measure the deep body temperature. Mice restrained in a cylinder with a diameter of 25–35 mm showed a marked reduction in rectal temperature compared with controls (Fig. 1G; Control: 37.36 ± 0.15 °C, *n* = 8, 25 mm: 35.43 ± 0.33 °C, *n* = 6, *P* < 0.001; 30 mm: 35.97 ± 0.22 °C, *n* = 6, *P* = 0.002; 35 mm: 36.10 ± 0.31 °C, *n* = 9, *P* = 0.002), whereas 40- and 45-mm cylinders showed no significant difference from controls (40 mm: 37.07 ± 0.20 °C, *n* = 6, *P* > 0.999; 45 mm: 37.19 ± 0.13 °C, *n* = 6, *P* > 0.999). When using a cylinder with a diameter of 25–35 mm, a decrease in rectal temperature was observed even at the 60-min time point (Fig. S2).

Therefore, in our experimental system, when using a cylinder with a diameter of 25–35 mm, a larger and more sustained decrease (lasting over 45 min) in rectal temperature was observed compared with the decrease in body surface temperature.

### Both vertical and horizontal restrictions of movement caused a decrease in rectal temperature

**Figure 2:**
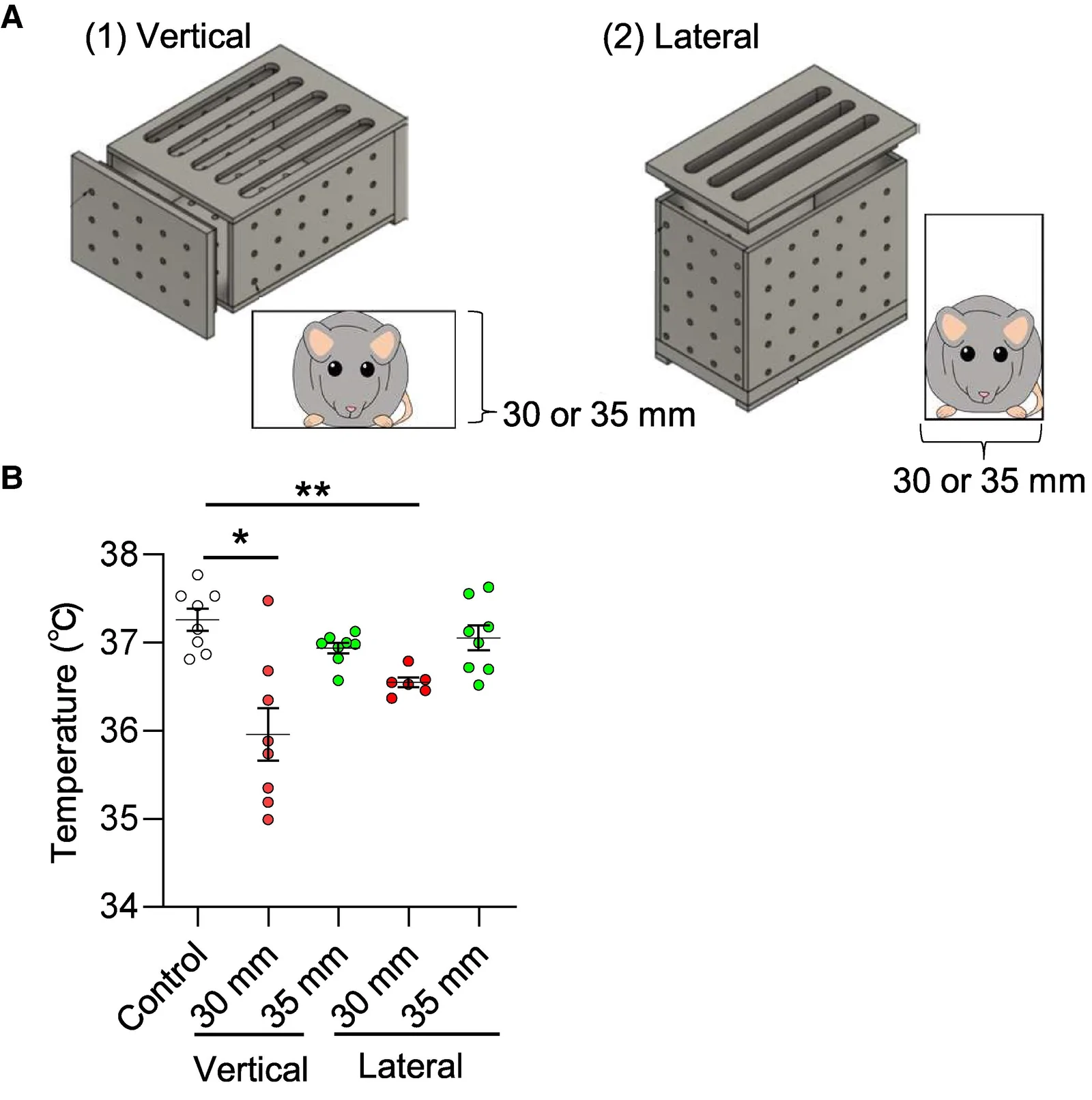
Both vertical and horizontal movement restrictions induced a decrease in rectal temperature. A) 100 mL rectangular containers were designed with a constant length (63 mm) and different heights or widths. Mice were restrained in the vertical direction with the containers having a height of 30 or 35 mm (1) or lateral direction with the containers having a width of 30 or 35 mm (2). B) Rectal temperatures were measured 15 min after the onset of the restraint. Statistical differences were determined using Dunnett's test following Welch's ANOVA (*P < 0.05, **P < 0.01).

Restraint using cylinders combines several elements: restriction of vertical body movement (i.e. ventral–dorsal direction), restriction of horizontal (lateral) body movement, and limitation of available space volume. We then investigated which physical restriction factors caused a decrease in rectal temperature due to restraint. We prepared rectangular containers with heights of 30 and 35 mm, both designed to have the same volume as a cylinder with a diameter of 45 mm (100 mL), which did not cause a significant decrease in rectal temperature (Fig. 1G), and the mice were placed inside them (Figs. 2A left and S3). Vertical restraint with the 30 mm container induced a modest but statistically significant reduction in rectal temperature compared to that of the controls (Fig. 2B; Control: 37.26 ± 0.12 °C, *n* = 8; 30 mm: 35.96 ± 0.30 °C, *n* = 8, *P* = 0.011), whereas the 35 mm container did not produce a significant change (36.94 ± 0.06 °C, *n* = 8; *P* = 0.142). Similarly, lateral restraint with the 30 mm wide container decreased the rectal temperature (Fig. 2A right, B, Fig. S3; 36.55 ± 0.06 °C, *n* = 6, *P* = 0.002), whereas the 35 mm wide container showed no significant difference from the controls (37.06 ± 0.14 °C, *n* = 8, *P* = 0.726).

These results indicate that both vertical and horizontal restrictions of movement can cause a decrease in rectal temperature. On the other hand, limitations on the available space volume do not appear to be the primary factor.

### Restraining only the limbs induced a marked decrease in the rectal temperature

**Figure 3:**
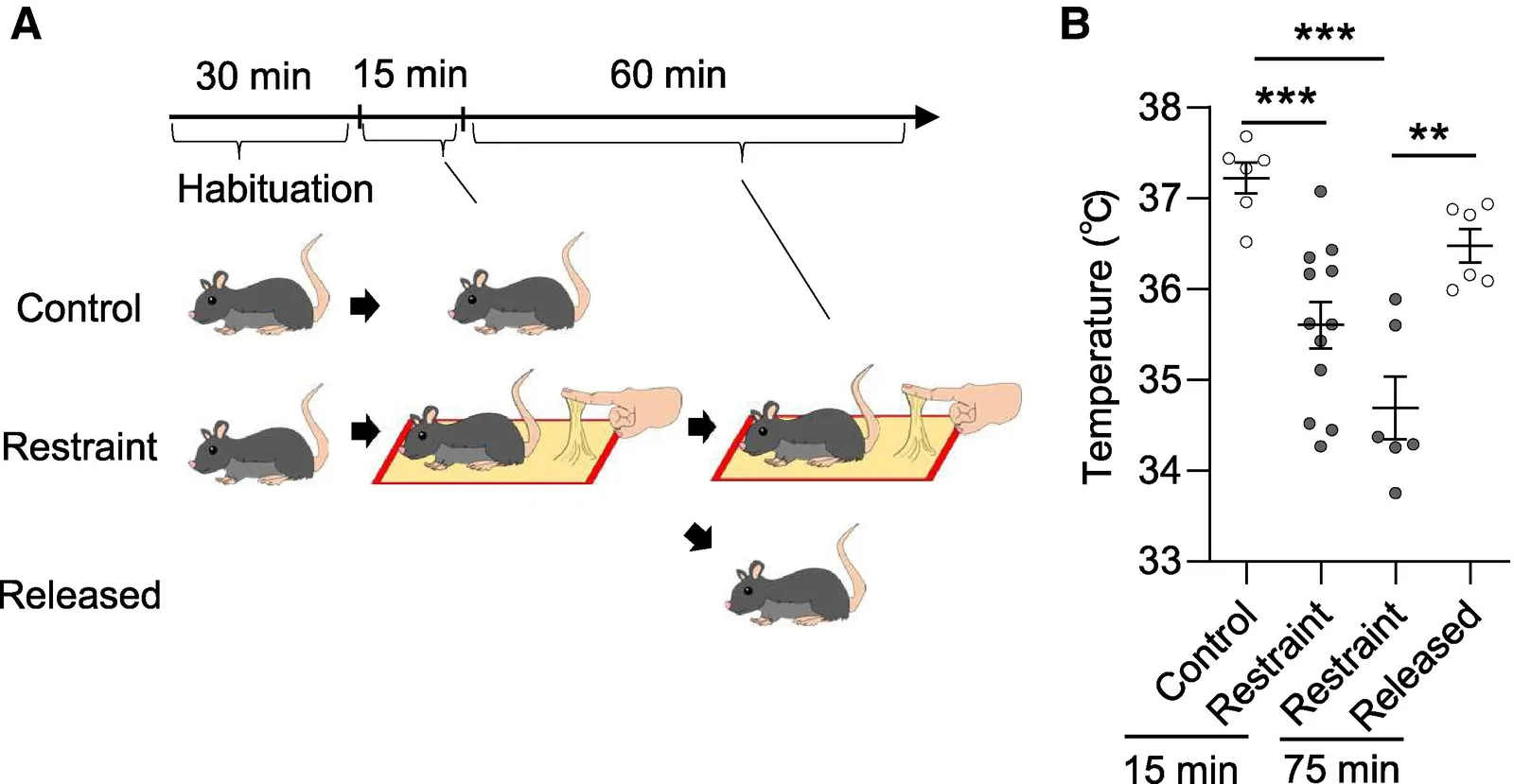
Restraint using glue traps caused a decrease in rectal temperature. A) After 30 min of habituation to the arena, the mice were fixed with glue traps for 15 min. Then, the mice were kept restrained or released for 60 min. B) Rectal temperatures were measured at 15 or 75 min after the onset of restraint. Statistical differences were determined by Bonferroni's test following one-way ANOVA (**P < 0.01, ***P < 0.001).

Next, we investigated which part of the body's movement restriction is crucial for lowering rectal temperature. In the experiment shown in Fig. 2, under conditions where a significant decrease in rectal temperature was observed, the mice's limb movements were more severely restricted than under conditions where no significant decrease was observed (Fig. S3). To test whether limb restraint induces hypothermia, mice were subjected to temporary limb restraint using glue traps coated with a sticky adhesive (Fig. 3A). Mice restrained for 15 min showed a significant decrease in rectal temperature compared to controls (Fig. 3B; Control: 37.23 ± 0.17 °C, *n* = 6; Restraint _(15min)_: 35.60 ± 0.26 °C, *n* = 12, *P* = 0.001). When released from glue traps after 15 min of restraint, the rectal temperature recovered to near the control level by 60 min (Fig. 3B; Released: 36.48 ± 0.18 °C, *n* = 6; Control vs. Released, *P* = 0.549). However, when restrained for an additional 60 min after the initial 15 min, the rectal temperature remained low (Fig. 3B; Restraint _(75min)_: 34.69 ± 0.35 °C, *n* = 6; Control vs. Restraint _(75min)_, *P* < 0.001; Restraint _(75min)_ vs. Released, *P* = 0.002).

**Figure 4:**
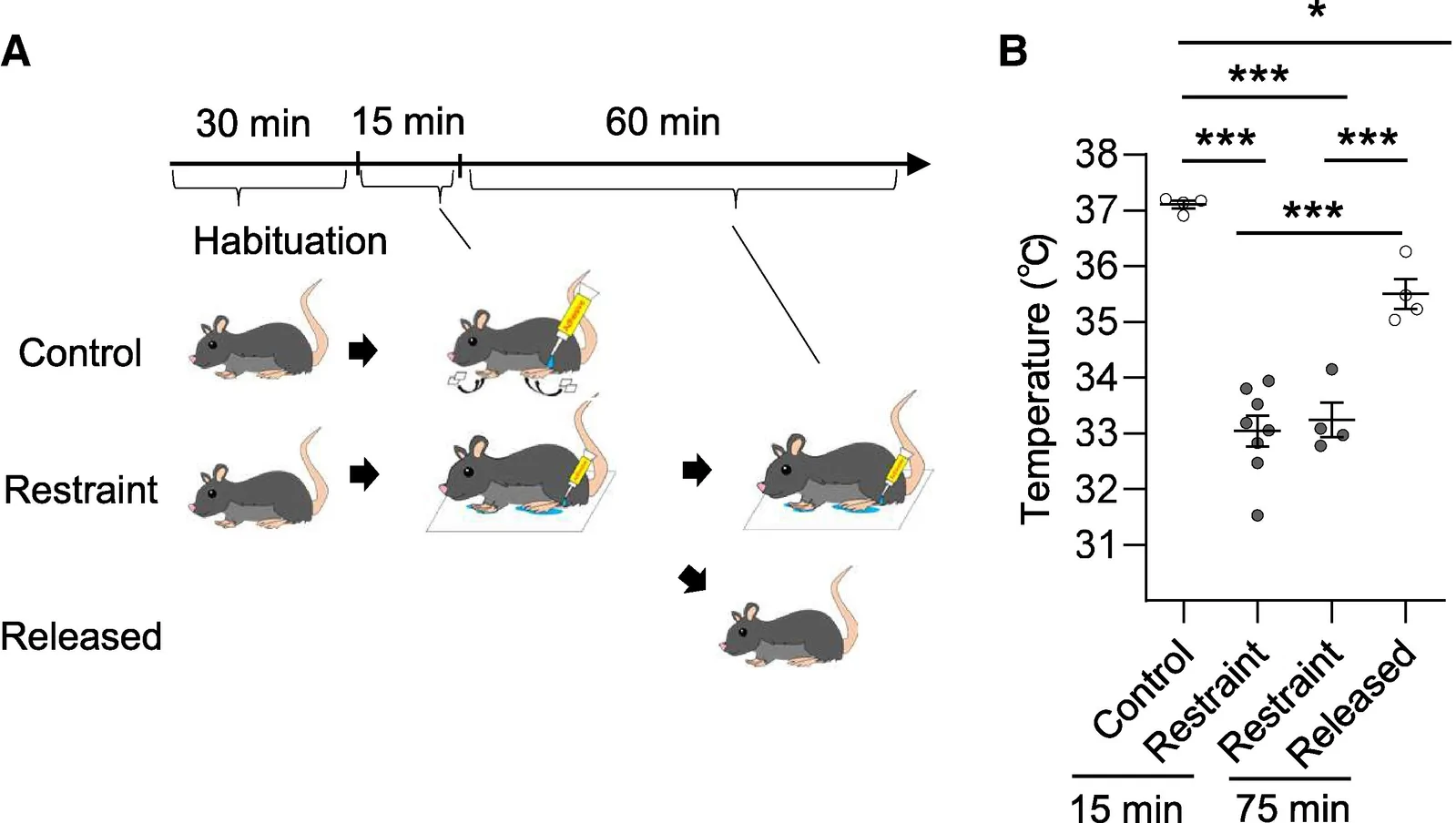
Fixation of the limbs to the board using cyanoacrylate adhesive induced a marked decrease in rectal temperature. A) Schematic of the experimental schedule. The control mice received sham operation, which attached 5 × 5 mm cardboard to each sole with adhesive, at time 0. In the restrained group, the soles of each mouse were fixed to 100 × 100 mm cardboard. Some restrained mice were released at 15 min. B) Rectal temperatures were measured at 15 or 75 min after the onset of restraint. Statistical differences were determined by Bonferroni's test following one-way ANOVA (*P < 0.05, ***P < 0.001).

When using the glue traps, adhesion was initially observed only between the limbs and the board, but over time, adhesion was also observed between the abdomen and the board in most (>80%) mice. Therefore, we conducted an additional experiment in which only the mouse limbs were fixed to the board using cyanoacrylate adhesive (Fig. 4A). In the restrained mouse group, the soles of the paws were fixed to a large piece of cardboard (100 × 100 mm) using adhesive. In the control group, the same adhesive was used to attach a small piece of cardboard (5 × 5 mm) to the soles, allowing the limbs to move to some extent. Mice restrained by their limbs on the large board for 15 min showed a robust reduction in rectal temperature compared with the controls (Fig. 4B; Control: 37.11 ± 0.07 °C, *n* = 4; Restraint _(15min)_: 33.04 ± 0.28 °C, *n* = 8, *P* < 0.001). Upon release from the board after 15 min of restraint, the rectal temperature increased but did not recover to the control level within 60 min (Fig. 4B; Released: 35.50 ± 0.27 °C, *n* = 4; Restraint _(15min)_ vs. Released, *P* < 0.001; Control vs. Released, *P* = 0.015). On the other hand, when mice were restrained for an additional 60 min after the initial 15 min, their rectal temperature remained extremely low (Fig. 4B; Restraint _(75min)_: 33.25 ± 0.31 °C, *n* = 4; Control vs. Restraint _(75min)_, *P* < 0.001; Restraint _(15min)_ vs. Restraint _(75min)_, *P* > 0.999). When the mouse's abdomen was fixed to a cardboard sheet using the same adhesive, no significant decrease in rectal temperature was observed (Fig. S4).

These results indicate that a marked decrease in rectal temperature can be induced by restraining only the limbs of the mice.

### The restriction of limb movement played a major role in the decrease in rectal temperature caused by the cylinder restraint

**Figure 5:**
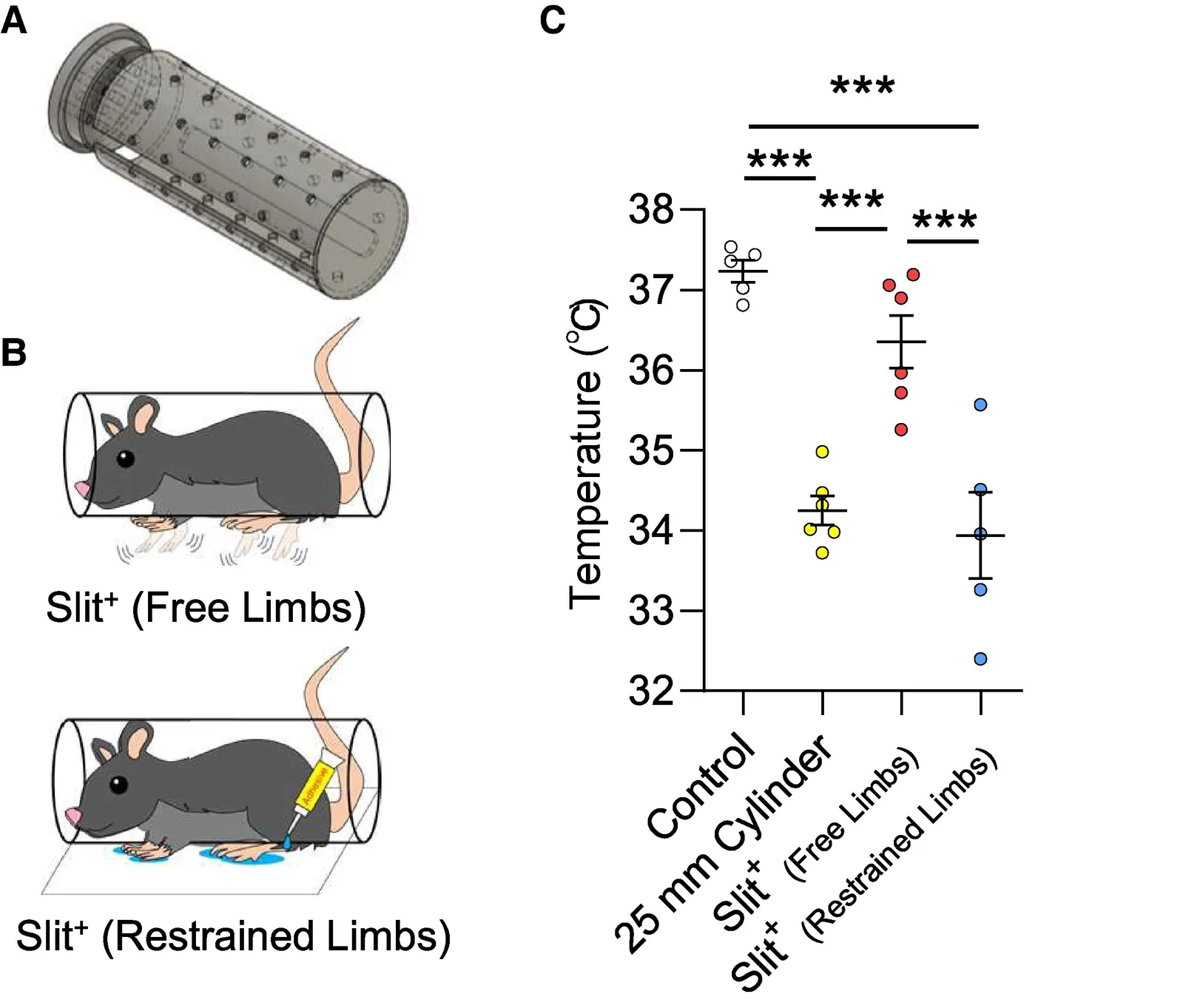
The decrease in rectal temperature under cylindrical restraint was rescued by allowing the mouse to move its limbs. A) To let the mice have their limbs out of the cylinder, a pair of 6 mm width slits were made in the Φ = 25 mm cylinder. B) The mice could let their limbs out through the slits and move them freely in the Slit^+^_(Free Limbs)_ group. In the Slit^+^_(Restrained Limbs)_ group, the limbs were attached to the cardboard sheet with adhesive. C) After 30 min of habituation, the mice were restrained in each condition for 15 min. Rectal temperature at 15 min after the onset of restraint is shown in the graph. Statistical differences were determined using Bonferroni's test following one-way ANOVA (***P < 0.001).

Next, we investigated the extent to which limb restraint contributed to the decrease in rectal temperature in the cylinder restraint experiment shown in Fig. 1G. To allow the mouse to move its limbs while inserted into a cylinder with a diameter of 25 mm, we created 6 mm wide slits on both sides of the cylinder and extended the mouse limbs through them. We then wrapped tape around the limbs to prevent the mice from retracting them inside the cylinder (Fig. 5A, B). When the mice were placed in the cylinder allowing limb movement, no significant decrease in rectal temperature was observed compared to the unconstrained control group (Fig. 5C; Control: 37.23 ± 0.14 °C, *n* = 5; Slit^+^  _(Free Limbs)_: 36.35 ± 0.33 °C, *n* = 6, *P* = 0.427). This contrasts with the marked decrease in rectal temperature observed when the mice were placed in a cylinder without slits (Fig. 5C; 25 mm Cylinder: 34.25 ± 0.18 °C, *n* = 6; Control vs. 25 mm Cylinder, *P* < 0.001; 25 mm Cylinder vs. Slit^+^  _(Free Limbs)_, *P* < 0.001). However, when mice were placed in cylinders with slits and their limbs protruding through the slits were fixed to a large cardboard sheet with adhesive, a comparable decrease in rectal temperature was observed as when placed in cylinders without slits (Fig. 5B, C; Slit^+^  _(Restrained limbs)_: 33.94 ± 0.54 °C, *n* = 5; Control vs. Slit^+^  _(Restrained limbs)_, *P* < 0.001; Slit^+^  _(Free limbs)_ vs. Slit^+^  _(Restrained limbs)_, *P* < 0.001; 25 mm Cylinder vs. Slit^+^  _(Restrained limbs)_, *P* > 0.999). These results once again demonstrate that restraining the limbs of mice alone can cause a marked decrease in rectal temperature and suggest that the restriction of limb movement plays a major role in the decrease in rectal temperature caused by cylinder restraint.

### In a state of limb restraint, activation of the HPA axis was suggested

Next, we examined whether the HPA axis, which is commonly activated when exposed to stressors, was activated in the limb-restrained state.

**Figure 6:**
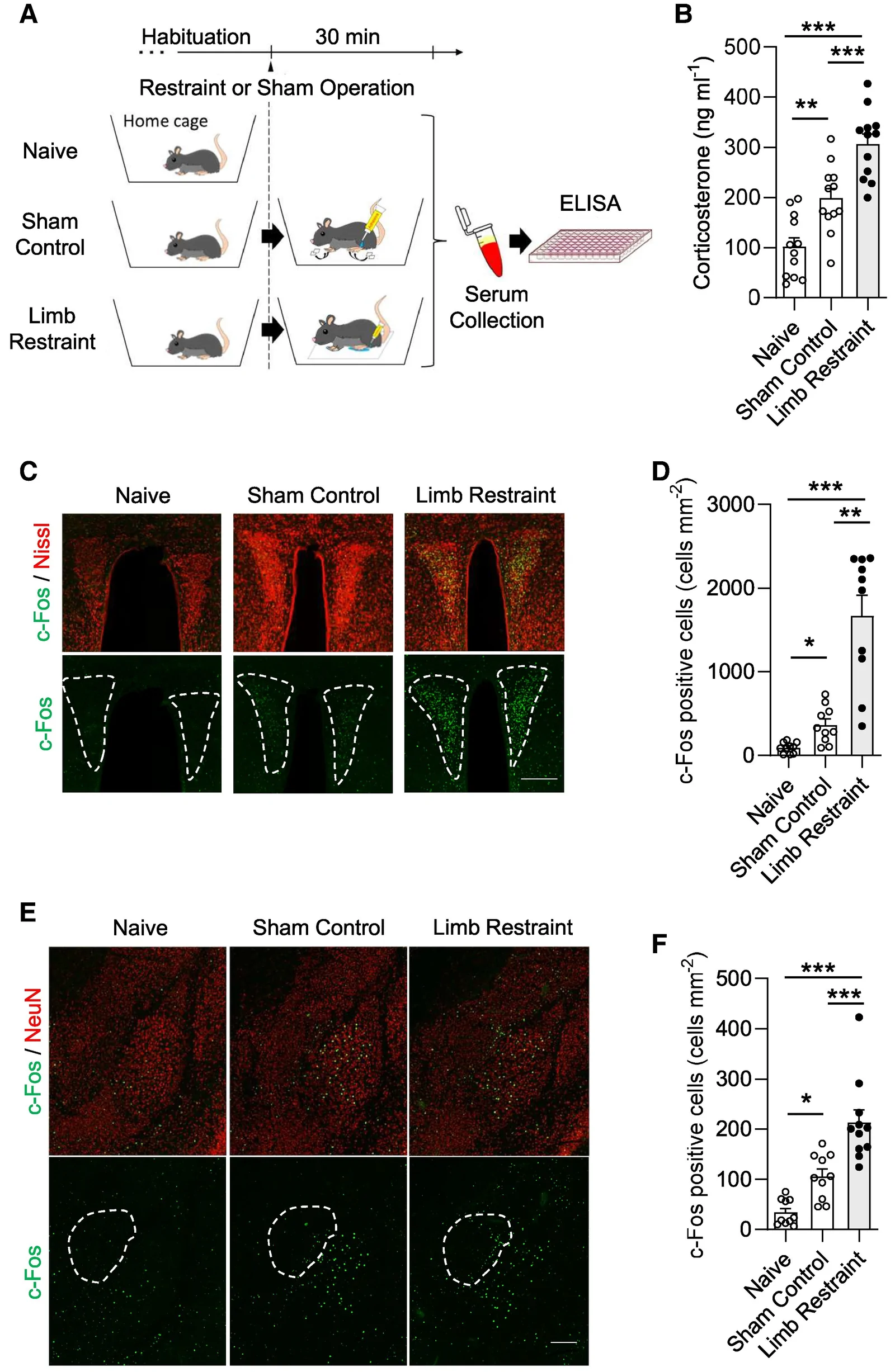
Activation of the HPA axis in limb restraint mice. A) Corticosterone concentrations were measured in serum of naïve, sham control, and limb restraint mice. Schematic of the experimental schedule is shown. Thirty minutes after beginning of restraint or sham operation, serum samples were collected, and corticosterone levels were measured by ELISA. The concentrations are presented in (B). C) Representative images of immunohistochemistry and Nissl staining around the PVN from the coronal brain section 0.7 mm posterior to bregma. The sections from naïve, sham control and limb restraint mice were stained with anti-c-Fos antibody (green in upper and lower images) and Nissl staining (red in upper images). In the lower images, the area of the PVN is surrounded by dashed lines. D) Quantitative analysis of c-Fos expression in the PVN. E) Representative images of immunohistochemistry around the amygdala from coronal brain section 1.3 mm posterior to bregma. The sections were stained with antibodies against c-Fos (green) and NeuN (red in upper images). The area of the CeA is surrounded by dashed lines. The area adjacent to the right of the region outlined by the dashed line contains the basolateral amygdala. F) Quantitative analysis of c-Fos expression in the CeA. Statistical differences were determined with Dunnett's test following Welch's ANOVA in (D) and Bonferroni's test following one-way ANOVA in (B, F) (*P < 0.05, **P < 0.01, ***P < 0.001). Scale bar, 200 μm.

The following three groups of mice were prepared: (i) naïve mice kept in their home cages, (ii) sham-control mice with small pieces of cardboard attached to the soles of their paws, and (iii) limb-restraint mice with the soles of their paws fixed to a large piece of cardboard (Fig. 6A). Then blood was collected from these mice, and serum corticosterone concentrations were measured by ELISA. Mice subjected to 30 min of restraint showed significantly higher levels of corticosterone than naïve or sham-control mice (Fig. 6B; Naïve: 102.1 ± 17.8 ng/mL, *n* = 12; Sham: 198.5 ± 19.4 ng/mL, *n* = 12; Limb Restraint: 306.6 ± 19.8 ng/mL, *n* = 12; Naïve vs. Limb Restraint, *P* < 0.001; Sham vs. Limb Restraint, *P* < 0.001). This increase in serum corticosterone levels appeared to contribute little to the decrease in rectal temperature, because administration of RU486, a glucocorticoid receptor and progesterone receptor antagonist, prior to restraint did not result in a significant difference in the decrease in rectal temperature compared to administration of the vehicle (Fig. S5).

Furthermore, c-Fos immunostaining of the collected brains revealed a marked increase in c-Fos-positive cells in the paraventricular hypothalamic nucleus (PVN), which plays a key role in the HPA axis, in limb-restrained mice compared with that in naïve or sham-operated control (Fig. 6C, D; Naïve: 93 ± 20 cells/mm^2^, *n* = 10; Sham: 364 ± 70 cells/mm^2^, *n* = 10; Limb Restraint: 1668 ± 245 cells/mm^2^, *n* = 10; Naïve vs. Limb Restraint, *P* < 0.001; Sham vs. Limb Restraint, *P* = 0.001), suggesting strong PVN activation during limb restraint. Moreover, c-Fos-positive cells also increased in the central amygdala (CeA), which is known to play a crucial role in PVN activation (Fig. 6E, F; Naïve: 33.9 ± 8.1 cells/mm^2^, *n* = 10; Sham: 105.8 ± 14.3 cells/mm^2^, *n* = 10; Limb Restraint: 213.2 ± 25.0 cells/mm^2^, *n* = 11; Naïve vs. Limb Restraint, *P* < 0.001; Sham vs. Limb Restraint, *P* < 0.001). Taken together, these results suggest that the HPA axis is activated in a state of limb restraint, similar to many stress responses accompanied by an increase in body temperature.

### Both suppressed heat production in the BAT and increased heat loss from the tail contributed to limb restraint-induced hypothermia

To obtain insight into the mechanisms underlying limb restraint-induced hypothermia, we then investigated heat production and heat dissipation in peripheral tissues during limb restraint.

**Figure 7:**
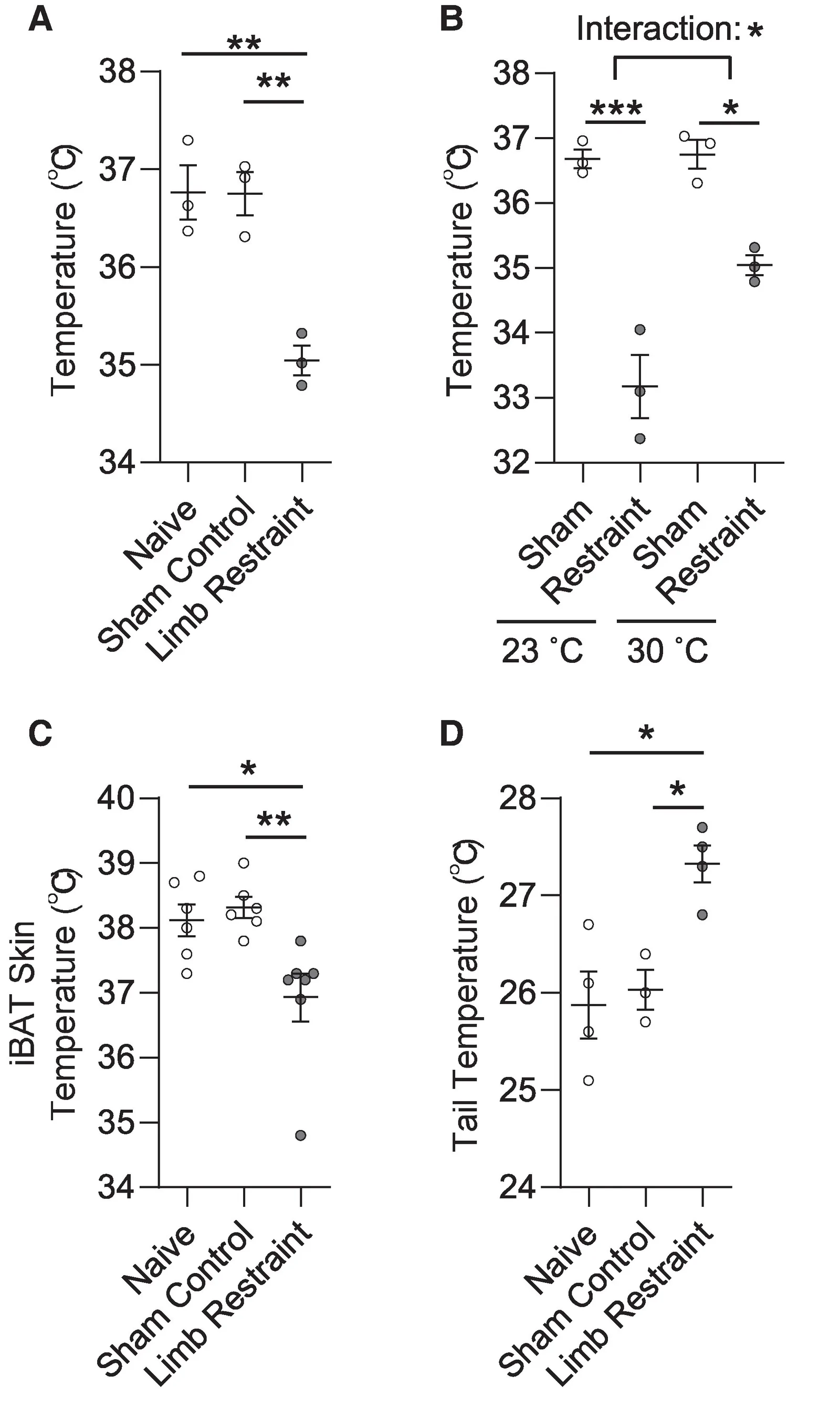
Suppressed heat production in the BAT and increased heat loss from the tail contributed to limb restraint-induced hypothermia. A, B) Rectal temperatures were measured in naïve, sham-control, and limb-restrained mice under ambient temperature conditions of 23 °C or 30 °C. Mice were acclimated to the ambient temperatures for more than 30 min before restraint, and the rectal temperatures were measured 15 min after the onset of restraint. The rectal temperatures measured at 30 °C are shown in (A). The rectal temperatures measured at 23 °C and 30 °C are compared in (B). C, D) The temperatures of the skin covering the iBAT and of the tail surface in naïve, sham-control, and limb-restrained mice are shown in (C) and (D), respectively. C) The iBAT skin temperatures were measured 15 min after the onset of restraint. D) The tail surface temperatures were measured 5 min after the onset of restraint. Statistical differences were determined using Bonferroni's test following one-way ANOVA in (A, C, D), and Bonferroni's test following two-way ANOVA in (B) (*P < 0.05, **P < 0.01, ***P < 0.001).

First, we examined whether the decrease in rectal temperature occurs even under thermoneutral conditions. When mice are kept at an ambient temperature of 20–23 °C, a state lower than their thermoneutrality (37), they maintain their Tc through cold-induced thermogenesis (which occurs primarily in BAT (38), and possibly, to some extent, in muscle and other tissues (39, 40)), but this thermogenesis can be nullified in a thermoneutral state of 30 °C. Mice were acclimated to an ambient temperature of 30 °C for more than 30 min, and then their limbs were restrained in the same 30 °C environment. Under this condition, limb restraint still induced a significant decrease in rectal temperature at 15 min, compared with naïve and sham-control mice (Fig. 7A; Naïve: 36.77 ± 0.28 °C, *n* = 3; Sham: 36.75 ± 0.22 °C, *n* = 3; Limb restraint: 35.04 ± 0.15 °C, *n* = 3; Naïve vs. Limb restraint, *P* = 0.005; Sham vs. Limb restraint, *P* = 0.005). However, the degree of decrease was smaller than when the experiment was conducted at 23 °C (Fig. 7B; Sham_(23°C)_: 36.68 ± 0.15 °C, *n* = 3; Limb restraint _(23°C)_: 33.17 ± 0.49 °C, *n* = 3; Sham _(23°C)_ vs. Limb restraint _(23°C)_, *P* < 0.001; Sham _(23°C)_ vs. Sham _(30°C)_, *P*  *>* 0.999; Limb restraint _(23°C)_ vs. Limb restraint _(30°C)_, *P* = 0.011; *F*(1, 8) = 9.78, *P* = 0.014 for two-way ANOVA interaction). These results suggest that the rapid suppression of cold-induced thermogenesis partially contributes to the decrease in rectal temperature observed after limb restraint at 23 °C, while other mechanisms contribute to the full decrease in rectal temperature.

Next, we examined changes in the temperature of the skin covering the iBAT following limb restraint at 23 °C. Consistent with the findings described above, the temperature of the skin covering the iBAT showed a significant decrease 15 min after the start of restraint (Fig. 7C; Naïve: 38.12 ± 0.24 °C, *n* = 6; Sham: 38.32 ± 0.17 °C, *n* = 6; Limb restraint: 36.93 ± 0.37 °C, *n* = 7; Naïve vs. Limb restraint, *P* = 0.028; Sham vs. Limb restraint, *P* = 0.010).

Finally, we investigated the changes in the surface temperature of the tail, which is important for heat dissipation in mice. When limb restraint was performed at 23 °C, a small but significant increase in temperature was observed 5 min after the start of restraint (Fig. 7D; Naïve: 25.88 ± 0.34 °C, *n* = 4; Sham: 26.03 ± 0.20 °C, *n* = 3; Limb restraint: 27.33 ± 0.19 °C, *n* = 4; Naïve vs. Limb restraint, *P* = 0.012; Sham vs. Limb restraint, *P* = 0.033).

In summary, these results suggest that the decrease in rectal temperature observed following limb restraint at 23 °C was at least partially attributable to a combination of suppressed heat production in the BAT and increased heat loss from the tail.

## Discussion

In this study, we demonstrated that in mice, the simple procedure of limb restraint reliably induces a rapid (within 15 min) and substantial (∼4 °C) decrease in rectal temperature, which is a good indicator of Tc. Previous studies using conventional cylindrical or mesh-based restraint paradigms have reported heterogeneous outcomes, with some showing hyperthermia (27–30) and others hypothermia (17, 18, 20, 21). These differences in results have generally been attributed to the species, strain, and age of the experimental animals used, as well as the characteristics of the restraint equipment employed in the studies. However, our results suggest that the degree of limb movement restriction is also an important factor influencing outcomes within the restraint paradigm. In particular, the results in Fig. 5 highlight the significant contribution of limb movement restriction to the reduction in Tc. In this experiment, mice were placed in cylinders with a diameter of 25 mm, and their limbs were positioned to extend through the slits. They were then divided into two groups: one in which the limbs protruding through the slits could move freely, and another in which the limbs were immobilized. In the former group, no significant decrease in rectal temperature was observed, whereas in the latter group, a marked decrease in rectal temperature was noted (Fig. 5C). There was no difference in the contact between the cylinder and the mouse body between the former and latter groups. Therefore, it is considered that the effects of direct contact between the cylinder and the body, or the effects of the cylinder compressing the mouse's body—including restricted breathing—did not significantly influence the decrease in rectal temperature. However, the difference in whether or not to restrict limb movement through the slits resulted in a significant difference in rectal temperature, suggesting that restricting limb movement is a major contributing factor to the decrease in rectal temperature.

Limb restraint induced a decrease in rectal temperature, while simultaneously inducing an increase in serum corticosterone concentration and activation of the PVN (Fig. 6A–D), suggesting HPA axis activation. This strongly suggests that a stress response involving Tc reduction was induced by limb restraint. Stressors are generally divided into physical and psychological stressors. While the former involves direct physical invasion that may threaten life and cause accompanying physical pain, the latter does not itself produce physical pain but is accompanied by anticipation of physical pain, discomfort, or fear (41). In our observations, when the mice's limbs were fixed to a board with adhesive, they vigorously moved their entire bodies in an attempt to break free for the first 1–2 min. However, after that, they remained essentially still, and such vigorous movements were observed only sporadically. Considering that no significant decrease in rectal temperature was observed when the mice's abdomens were fixed to the cardboard with adhesive (Fig. S4), it may be possible to interpret that the irritation to the skin from the adhesive and the pain associated with movements to escape the restraint did not result in a significant change in rectal temperature. On the other hand, immobilizing the limbs is an action that deprives the mouse of any chance to escape external attacks, potentially having a significant impact on its survival. This likely caused the mice to experience intense discomfort and fear accompanied by amygdala activation. Therefore, limb restraint is considered to possess a strong nature as a psychological stressor. Interestingly, social defeat, which is classified within the same category of psychological stressors, is known to induce an increase in Tc (5, 6), contrary to that induced by limb restraint. Where the pathways diverge within the central nervous system remains to be clarified.

Regarding the mechanisms involved in restraint-induced hypothermia, a few reports using pharmacological experimental methods in rats suggest that the brain's dopaminergic and serotonergic signaling are important (19, 20), but the specific neural circuits involved remain unclear. On the other hand, the mechanisms underlying Tc reduction caused by high ambient temperature, innate fear odors, and hypergravity have been elucidated in recent years (14, 26, 42). These studies have shown that when hypothermia was induced, either or both of the following occurred: suppression of BAT-dependent heat production and facilitation of heat dissipation from glabrous skin such as the tail through vasodilation (14, 26, 43). Furthermore, these studies have shown that these mechanisms are controlled by premotor neurons in the rostral raphe pallidus nucleus (rRpa) and the rostral ventrolateral medulla (RVLM) via the sympathetic nervous system (44, 45), and that the neural circuits upstream of the premotor neurons in the rRpa and RVLM differ significantly in each case. In this study, we demonstrated that even with limb restraint, the suppression of iBAT heat production and the increase in heat dissipation from the tail contribute, at least partially, to the induction of hypothermia. However, the neural circuits involved in these responses remain unknown at this time, and it is important to elucidate them in the future. In the paradigm we proposed, where mouse limbs are fixed to cardboard with adhesive, factors such as contact between the restraint equipment and the body trunk or compression of the body trunk by the restraint equipment—which occur in conventional cylindrical or mesh-based restraint experiments—are eliminated. Consequently, the sensory afferent inputs that initiate the neural circuitry causing hypothermia are thought to originate primarily from superficial and/or deep sensations in the limbs. When combined with chemogenetic and optogenetic analytical techniques, this simplified paradigm will serve as a useful tool for elucidating the neural circuits responsible for restraint-induced hypothermia.

## Supplementary Material

pgag301_Supplementary_Data

## Data Availability

Data used in the study are included in the manuscript and/or supporting information.
