# Supplementary material for: Limb restraint induces a rapid and profound decrease in core body temperature associated with the stress response in mice
Source: PNAS Nexus. 2026 Sep 4;5(9):pgag301. doi: 10.1093/pnasnexus/pgag301 (PMC13596739; doi:10.1093/pnasnexus/pgag301)
Supplement: Supplementary file 1 [file pgag301_supplementary_data.zip › PNASNEXUS-PNASNEXUS-2026-00345RRR-s01.pdf]

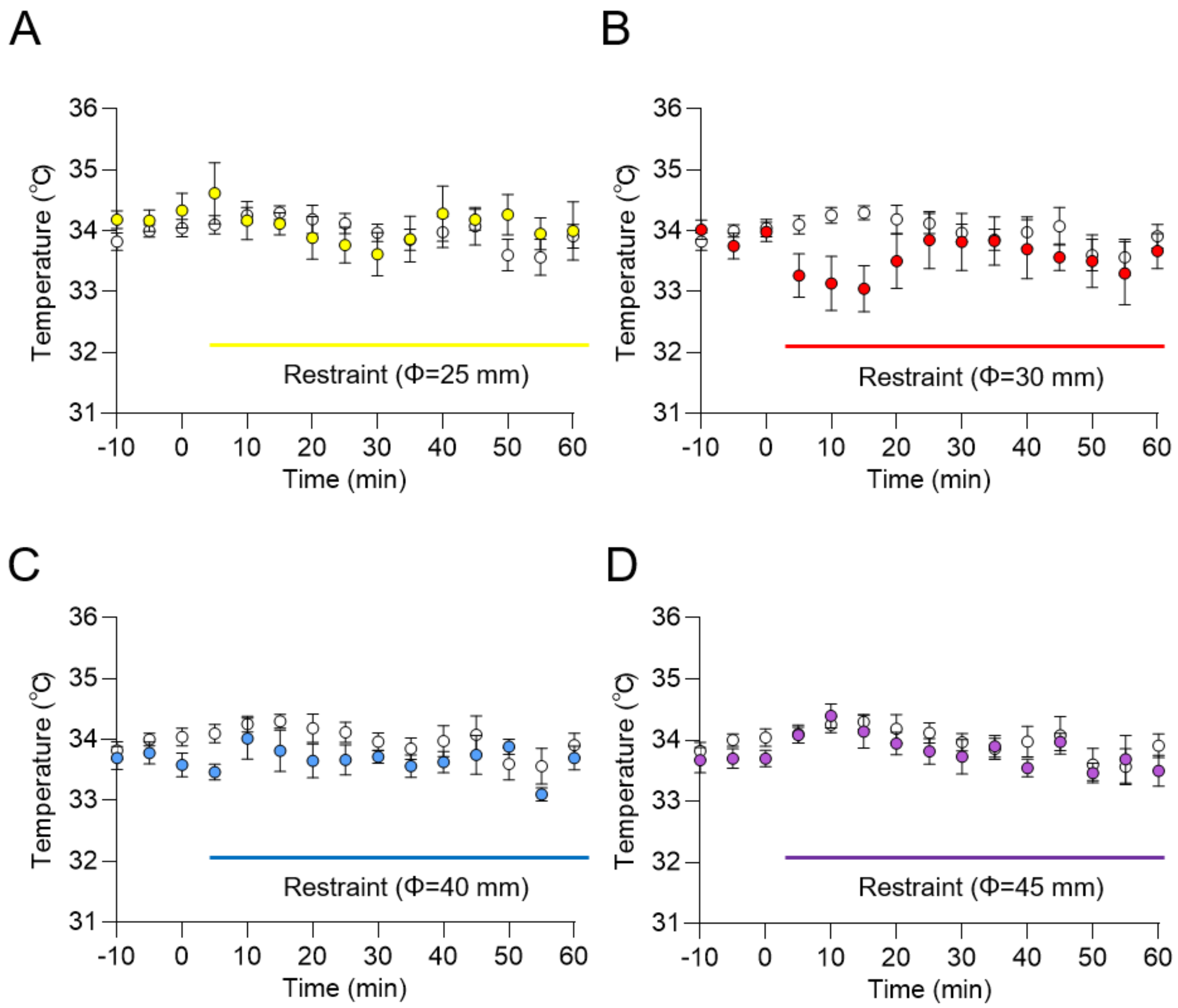

Supplemental Figure 1,

Same as Figure 1 E, changes in body surface temperature of mice were monitored during cylindrical restraint with Φ = 25-, 30-, 40-, and 45-mm cylinders. These temporal changes are plotted as colored circles in (A) to (D). Data from control conditions (open circles) are shown for comparison, as in Figure 1 E.

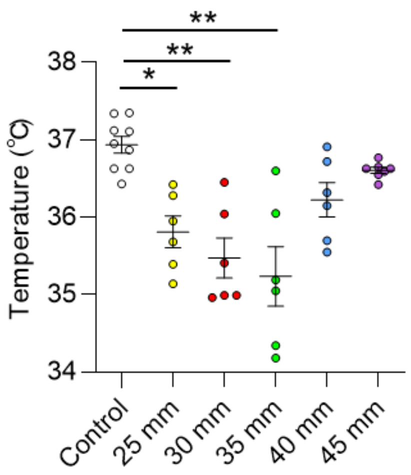

Supplemental Figure 2,

Rectal temperatures were measured at 60 min. Statistical differences were determined using Dunnett's test following Welch's ANOVA (* $P < 0.05$, ** $P < 0.01$).

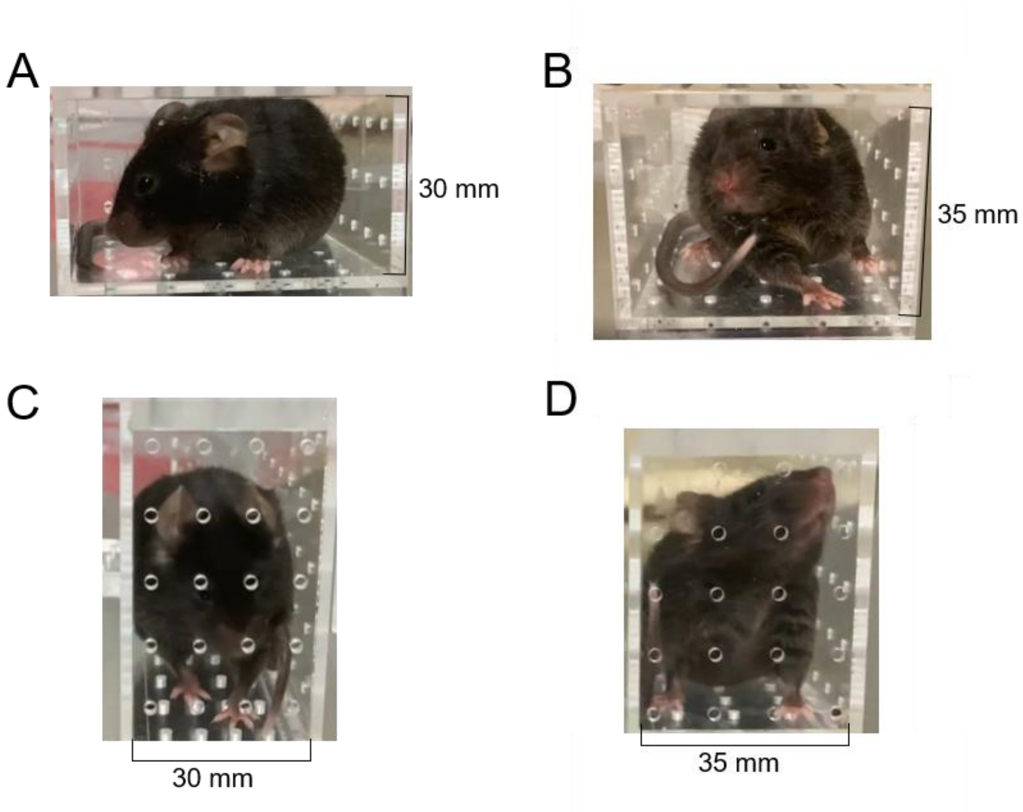

Supplemental Figure 3,

Representative images of mice when vertical body movement was restricted by placing them in rectangular containers 30 mm or 35 mm high (A, B), and when horizontal body movement was restricted by placing them in rectangular containers 30 mm or 35 mm wide (C, D).

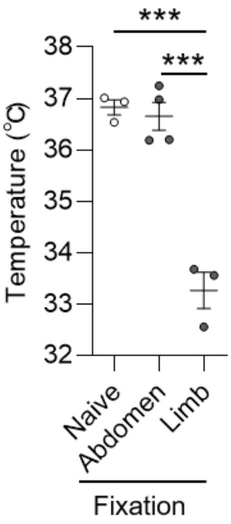

Supplemental Figure 4,

The abdomen or soles of mice were fixed to 100 × 100 mm cardboard using adhesive. The rectal temperatures of these mice and naïve mice were measured at 15 min after the onset of restraint. Statistical differences were determined by Bonferroni's test following One-way ANOVA (*** $P < 0.001$).

Naïve: 36.83 ± 0.15 °C, n = 3; Abdomen adhesion: 36.66 ± 0.27 °C, n = 4; Limb restraint: 33.27 ± 0.36 °C, n = 3; Naïve vs. Limb restraint, $P < 0.001$; Abdomen adhesion vs. Limb restraint, $P < 0.001$; Naïve vs. Abdomen adhesion, $P > 0.999$.

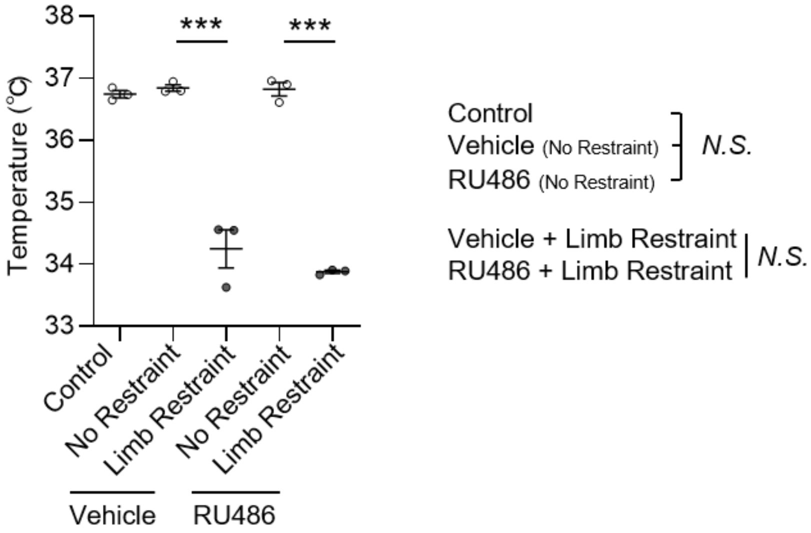

Supplemental Figure 5,

RU486 (10 μg/μl in 10% DMSO/sesame oil), vehicle (10% DMSO/sesame oil), or sesame oil alone (: control) was administered 1 h before restraint. Then, mice were either subjected to limb restraint or left undisturbed. Rectal temperatures were measured at 15 min after the onset of restraint. Statistical differences were determined by Bonferroni's test following One-way ANOVA (*** $P < 0.001$, *N.S.* not significant).

Control: 36.74 ± 0.06 °C, n = 3; Vehicle $_{\text{(no restraint)}}$: 36.85 ± 0.05 °C, n = 3; RU486 $_{\text{(no restraint)}}$: 36.82 ± 0.11 °C, n = 3; Vehicle + Limb restraint: 34.25 ± 0.31 °C, n = 3; RU486 + Limb restraint: 33.88 ± 0.02 °C, n = 3; Control vs. Vehicle $_{\text{(no restraint)}}$, $P > 0.999$; Control vs. RU486 $_{\text{(no restraint)}}$, $P > 0.999$; Vehicle $_{\text{(no restraint)}}$ vs. RU486 $_{\text{(no restraint)}}$: $P > 0.999$; Vehicle $_{\text{(no restraint)}}$ vs. Vehicle + Limb restraint, $P < 0.001$; RU486 $_{\text{(no restraint)}}$ vs. RU486 + Limb restraint, $P < 0.001$; Vehicle + Limb restraint vs. RU486 + Limb restraint, $P > 0.999$.
